# ﻿Asian species of the little-known subgenus Lagria (Lagriella) (Coleoptera, Tenebrionidae, Lagriinae), with a new species and the first record from China

**DOI:** 10.3897/zookeys.1265.171898

**Published:** 2025-12-30

**Authors:** Yong Zhou, Bin Chen

**Affiliations:** 1 Institute of Entomology and Molecular Biology, College of Life Sciences, Chongqing Normal University, Chongqing 401331, China Chongqing Normal University Chongqing China

**Keywords:** First record, identification key, Lagriini, lectotype, long-jointed beetle, Oriental region, taxonomy

## Abstract

The little-known subgenus Lagria (Lagriella) Borchmann, 1916 is characterized by pronounced sexual dimorphism, notably manifested in apterous females. In this paper, the Asian species of Lagria (Lagriella) are reviewed, including three known species, Lagria (Lagriella) bimarginata Fairmaire, 1896, L. (Lagriella) andrewesi Borchmann, 1916, L. (Lagriella) mima Borchmann, 1916, and the newly described L. (Lagriella) cordata**sp. nov.** from Guangxi, Guizhou, Hong Kong, Hunan, China. The new species represents the first record of the subgenus in China. Lectotypes are designated for L. (Lagriella) andrewesi and L. (Lagriella) mima. Habitus images, key diagnostic characters, ecological habitats of the new species, and type habitus of three known Asian species are documented. An identification key to the four Asian species is also provided.

## ﻿Introduction

The tribe Lagriini Latreille, 1825 comprises 135 extant genera (Bouchard et al. 2021; Aalbu et al. 2023). Hindwing reduction has not been documented in any of the twenty genera described since Borchmann’s (1936) monograph (Pic 1936, 1952a, 1952b, 1954, 1955; Borchmann 1942; Merkl 1987, 1988a, 1988b, 1991; Masumoto 1988; Chen 1997; Chen and Yuan 1996), nor in the two additional subgenera established within *Lagria* Fabricius, 1775 (Pic 1940; Borchmann 1942). Consequently, within the entire tribe, only three taxa are currently known to exhibit apterous conditions: the genus *Physolagria* Fairmaire, 1891, and the females of the subgenera Lagria (Apteronympha) Seidlitz, 1898 and Lagria (Lagriella) Borchmann, 1916 (Borchmann 1936).

As one of only two subgenera with apterous females within Lagriini, Lagria (Lagriella) can be distinguished from Lagria (Apteronympha) by the combination of two characters: the elytral apices of the female are distinctly protruding, and the terminal segment of the antennae is short. Borchmann (1936) further subdivided Lagria (Lagriella) into the African species group and the Asian species group. The Asian group comprises three species which are all recorded from southern India: L. (Lagriella) bimarginata Fairmaire, 1896 (Madurai), L. (Lagriella) andrewesi Borchmann, 1916 (Nilgiri Hills), and L. (Lagriella) mima Borchmann, 1916 (Nilgiri Hills). Few descriptions or records have resulted in the subgenus Lagria (Lagriella) remaining a little-known group.

After over a century, Lagria (Lagriella) cordata sp. nov., a small-sized species from China, is described as the fourth member of the Asian species group. This species also represents the first record of the subgenus in China and the third subgenus of *Lagria* to be recorded in the country. All four Asian species are reviewed and keyed here, with their habitus and key diagnostic features illustrated.

## ﻿Material and methods

The acronyms of depositories for specimens examined are as follows:

**CNU**Chongqing Normal University, Chongqing, China;

**IZCAS**Institute of Zoology, Chinese Academy of Sciences, Beijing, China;

**MNHN**Muséum national d’Histoire naturelle, Paris, France;

**ZMH**Museum of Nature Hamburg, Leibniz Institute for the Analysis of Biodiversity Change (formerly Zoological Museum Hamburg), Hamburg, Germany.

Photographs of the type specimens of L. (Lagriella) andrewesi, L. (Lagriella) mima, along with five additional male specimens of L. (Lagriella) bimarginata were kindly provided by ZMH; the female type photographs of L. (Lagriella) bimarginata were sourced from MNHN. Morphological examinations and dissections were conducted using a stereomicroscope (Olympus SZ2-ILST). Specimen habitus, diagnostic characters, and measurements were documented with a digital stereomicroscope (KEYENCE VHX-5000). Images were processed, annotated, and compiled into plates using ADOBE PHOTOSHOP CS6. Label data are presented verbatim in quotation marks (“”), with author interpretations enclosed in square brackets ([]). Line breaks on labels are indicated by a single slash (/).

## ﻿Results

### Subgenus﻿ Lagriella

Taxon classificationAnimaliaColeopteraTenebrionidae

Borchmann, 1916

0BA4EC84-3BDB-5B1E-8905-DFC4C4E99B07

Lagriella Borchmann, 1916: 90; Borchmann, 1936: 53 (brief description).

#### Distribution.

Afrotropical region, Indo-Malayan region.

### ﻿Key to Asian species of Lagria (Lagriella)

**Table:** 

1	Elytra bearing a dark longitudinal band medial to the humeral callosity (Fig. 4A, C); epipleura strongly widened (Fig. 4B, D); female elytra exceptionally convex (Fig. 5B), abruptly dilated in the basal 1/3 and forming a heart-shaped outline when closed (Fig. 4C)	**China (Guangxi, Guizhou, Hong Kong, Hunan). L. (Lagriella) cordata sp. nov.**
–	Elytra unicolorous, lacking dark band; epipleura not widened; female elytra moderately convex (Fig. 5C, D), gradually expanded posteriorly in basal 2/3	**2**
2	Dorsum with short setae (Fig. 2); antennomeres slender, male antennomere XI equal to combined length of preceding four segments (Borchmann 1936); female elytra slightly expanded posteriorly	**India (Tamil Nadu). L. (Lagriella) bimarginata**
–	Dorsum with long setae (Figs 1, 3); antennomeres short, male antennomere XI shorter than combined length of preceding four segments; female elytra moderately expanded posteriorly	**3**
3	Male elytra strongly expanded posteriorly	**India (Nilgiri Hills). L. (Lagriella) andrewesi**
–	Male elytra slightly expanded posteriorly	**India (Nilgiri Hills). L. (Lagriella) mima**

### ﻿ Lagria (Lagriella) andrewesi

Taxon classificationAnimaliaColeopteraTenebrionidae

Borchmann, 1916

7E432529-3ECE-55F7-9E84-FAAA141C8520

Fig. 1

Lagria andrewesi Borchmann, 1916: 91 (“Nilgiri-Hills”, description); Borchmann 1936: 53 (same as the previous).

#### Type material examined.

**Figure 1.**
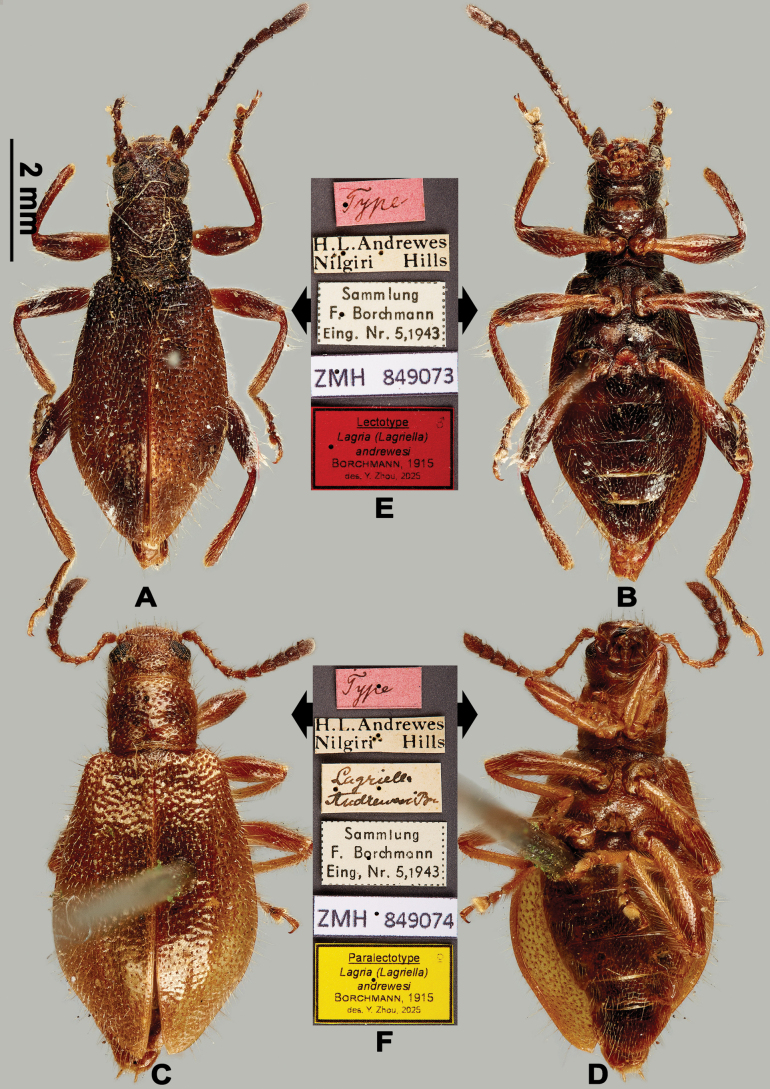
Habitus of L. (Lagriella) andrewesi. **A, B.** Lectotype: **A.** Dorsal view; **B.** Ventral view; **C, D.** Female paralectotype: **C.** Dorsal view; **D.** Ventral view; **E, F.** Labels: **E.** Labels of lectotype; **F.** Labels of paralectotype. Scale bar: 2 mm (**A–D**).

(1 ♂ 1 ♀). Lectotype, herewith designated. ***Lectotype***: ♂ (Fig. 1A, B): 1) “Type”, 2) “H. L. Andrewes / Nilgiri Hills”, 3) “Sammlung / F. Borchmann / Eing. Nr. 5, 1943”, 4) “ZMH 849073”, 5) “Lectotype ♂/ Lagria (Lagriella) / *andrewesi* / Borchmann, 1915 / des. Y. Zhou, 2025 [printed on red paper]”; ***Paralectotype***: 1 ♀ (Fig. 1C, D): 1) “Type”, 2) “H. L. Andrewes / Nilgiri Hills”, 3) “*Lagriella* / *andrewesi* Bm”, 4) “Sammlung / F. Borchmann / Eing. Nr. 5, 1943”, 5) “ZMH 849074”, 6) “Paralectotype ♀ / Lagria (Lagriella) / *andrewesi* / Borchmann, 1915 / des. Y. Zhou, 2025 [printed on yellow paper]”.

#### Diagnosis.

Brown, dorsal surface with long setae. Male hindwings well-developed, antennomere XI as long as the two preceding segments together, pronotum almost as long as wide, elytra moderately expanded in basal 2/3, femur moderately dilated; female apterous, antennomere XI shorter than the combined length of two preceding antennomeres.

#### Distribution.

India.

### ﻿ Lagria (Lagriella) bimarginata

Taxon classificationAnimaliaColeopteraTenebrionidae

Fairmaire, 1896

231736CA-9CCB-5027-95E0-818DA3B40372

Fig. 2

Lagria bimarginata Fairmaire, 1896: 40 (“Madura” [Madurai, Tamil Nadu, India], 1 female, description); Borchmann 1916: 91 (“Vorder-Indien”, redescription); Borchmann 1936: 53 (almost same as the previous).

#### Type material examined.

**Figure 2.**
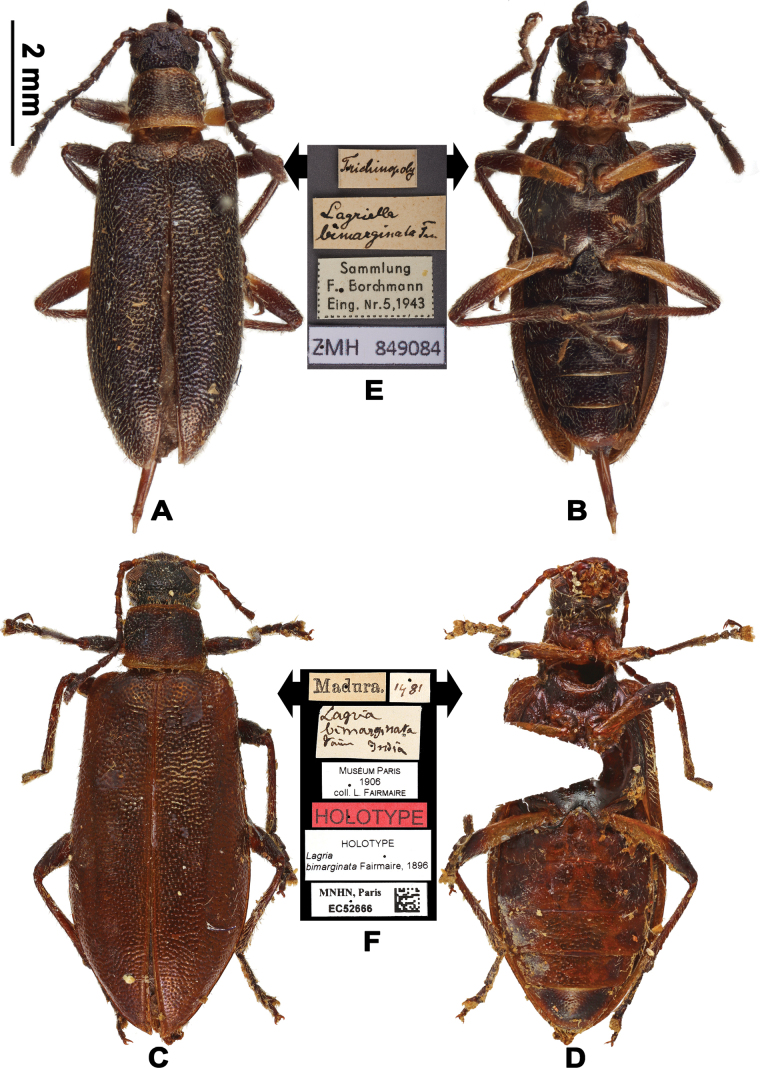
Habitus of L. (Lagriella) bimarginata. **A, B.** Male specimen determined by Borchmann: **A.** Dorsal view; **B.** Ventral view; **C, D.** Female holotype: **C.** Dorsal view; **D.** Ventral view; **E, F.** Labels: **E.** Labels of male specimen determined by Borchmann; **F.** Labels of holotype. Scale bar: 2 mm (**A–D**).

(1 ♀). ***Holotype***: ♀ (Fig. 2C, D): 1) “Madura”, 2) [1481? illegible handwritten text], 3) “*Lagria* / *bimarginata* / Fair India”, 4) MUSÉUM PARIS / 1906 / coll. L. FAIRMAIRE, 5) HOLOTYPE, 6) HOLOTYPE / *Lagria / bimarginata* Fairmaire, 1896, 7) MNHN, Paris / EC52666, followed by a QR code.

#### Other material examined.

(5 ♂) . 1 ♂: 1) “Trichinopoly [now Tiruchirappalli]”, 2) “*Lagriella* / *bimarginata* Fair”, 3) “Sammlung / F. Borchmann / Eing. Nr. 5, 1943”, 4) “ZMH 849082”; 1 ♂: 1) “Shembaganur Süd. India”, 2) “*Lagriella / bimarginata* Fair”, 3) “Sammlung / F. Borchmann / Eing. Nr. 5, 1943”, 4) “ZMH 849083”; 1 ♂ (Fig. 2A, B): 1) “Trichinopoly [now Tiruchirappalli]”, 2) “*Lagriella / bimarginata* Fair”, 3) “Sammlung / F. Borchmann / Eing. Nr. 5, 1943”, 4) “ZMH 849084”; 1 ♂: 1) “Trichinopoly [now Tiruchirappalli]”, 2) “Sammlung / F. Borchmann / Eing. Nr. 5, 1943”, 3) “ZMH 849085”; 1 ♂: 1) “Shembaganur Süd. India”, 2) “Sammlung / F. Borchmann / Eing. Nr. 5, 1943”, 3) “ZMH 849086”.

#### Diagnosis.

Brown, dorsal surface with short setae. Male hindwings well-developed, antennomere XI as long as the combined length of four preceding segments, pronotum as long as wide (Borchmann 1936); female apterous, antennomeres XI equal to the combined length of two preceding antennomeres, pronotum transverse, elytra slightly expanded in basal 2/3.

#### Distribution.

India.

### ﻿ Lagria (Lagriella) mima

Taxon classificationAnimaliaColeopteraTenebrionidae

Borchmann, 1916

A62EE22D-9F45-5EDF-B0E5-D88AD7C5C2DB

Fig. 3

Lagria mima Borchmann, 1916: 91 (“Nilgiri-Hills”, description); Borchmann 1936: 53 (same as the previous, habitus illustrations).

#### Type material examined.

**Figure 3.**
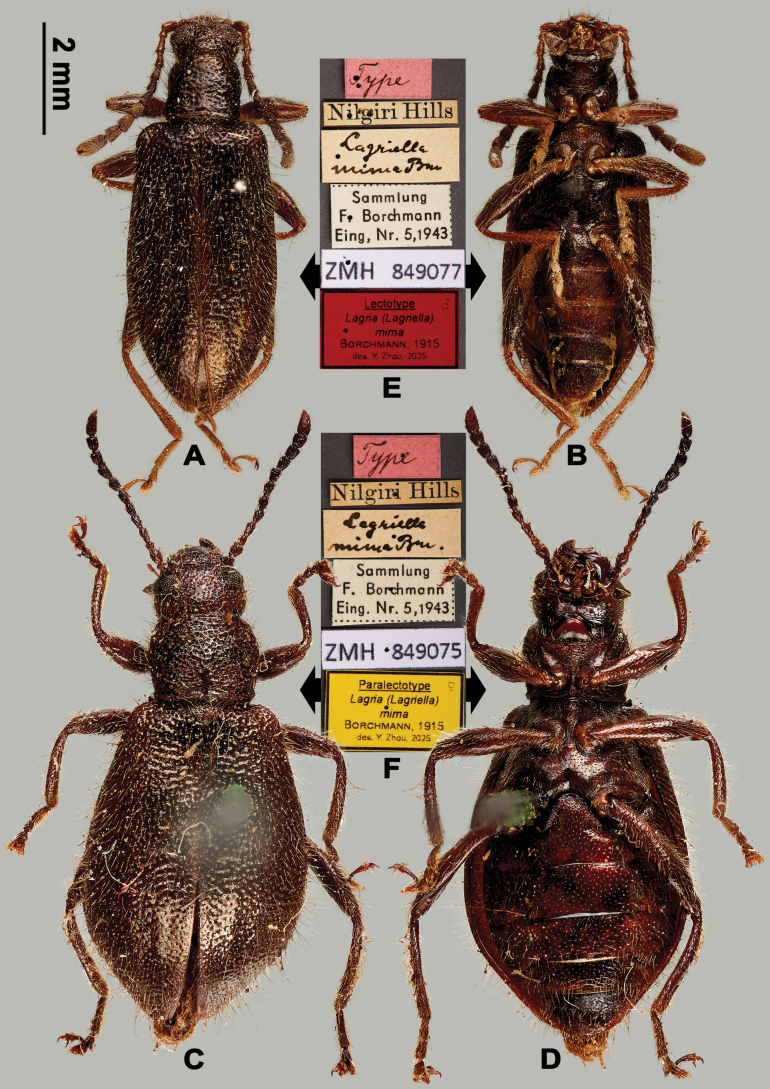
Habitus of L. (Lagriella) mima. **A, B.** Lectotype: **A.** Dorsal view; **B.** Ventral view; **C, D.** Female paralectotype (ZMH 849075): **C.** Dorsal view; **D.** Ventral view; **E, F.** Labels: **E.** Labels of lectotype; **F.** Labels of paralectotype. Scale bar: 2 mm (**A–D**).

(2 ♂ 2 ♀). Lectotype, herewith designated. ***Lectotype***: ♂ (Fig. 3A, B): 1) “Type”, 2) “Nilgiri Hills”, 3) “*Lagriella mima* Bm”, 4) “Sammlung / F. Borchmann / Eing. Nr. 5, 1943”, 5) “ZMH 849077”, 6) “Lectotype / Lagria (Lagriella) / *mima* / Borchmann, 1915 / des. Y. Zhou, 2025 [printed on red paper]”; ***Paralectotypes***: 1 ♀ (Fig. 3C, D): 1) “Type”, 2) “Nilgiri Hills”, 3) “*Lagriella mima* Bm”, 4) “Sammlung / F. Borchmann / Eing. Nr. 5, 1943”, 5) “ZMH 849075”, 6) “Paralectotype ♀ / Lagria (Lagriella) / *mima* / Borchmann, 1915 / des. Y. Zhou, 2025 [printed on yellow paper]”; 1 ♀: same as the previous, but “H. L. Andrewes / Nilgiri Hills”, “ZMH 849076”; 1 ♂: same as the previous, but “mima”, “ZMH 849078”.

#### Diagnosis.

Brown, dorsal surface with long setae. Male hindwings well-developed, antennomere XI as long as the two preceding segments together, pronotum almost as long as wide, elytra slightly expanded posteriorly, femur moderately dilated; female apterous, antennomere XI shorter than the combined length of two preceding antennomeres. Most similar to L. (Lagriella) andrewesi, but male elytra slightly expanded backwards, female pronotum with denser punctures.

#### Distribution.

India.

### ﻿ Lagria (Lagriella) cordata sp. nov.

Taxon classificationAnimaliaColeopteraTenebrionidae

7BDBC43A-A9E4-5D25-826D-24283005A878

https://zoobank.org/17D29356-6C8E-47DC-A875-8884215008D4

Figs 4, 5, 6, 7

#### Type locality.

China • Guizhou, Guiyang City, Baiyun District, Niuchang Buyi Ethnic Town.

#### Type material.

**Figure 4.**
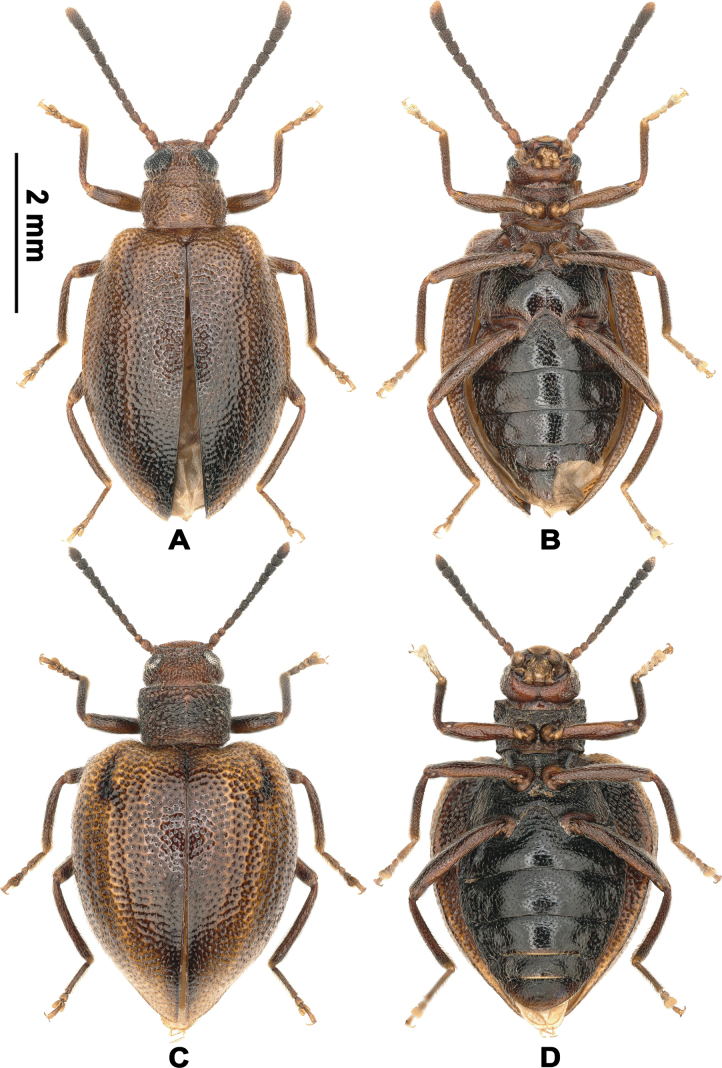
Habitus of L. (Lagriella) cordata sp. nov. **A, B.** Holotype: **A.** Dorsal view; **B.** Ventral view; **C, D.** Female paratype (2021.V.9, Guangxi, Hezhou City, Daguishan Mountain, Qixingchong, No. 21-5-9-1): **C.** Dorsal view; **D.** Ventral view. Scale bar: 2 mm (**A–D**).

**Figure 5.**
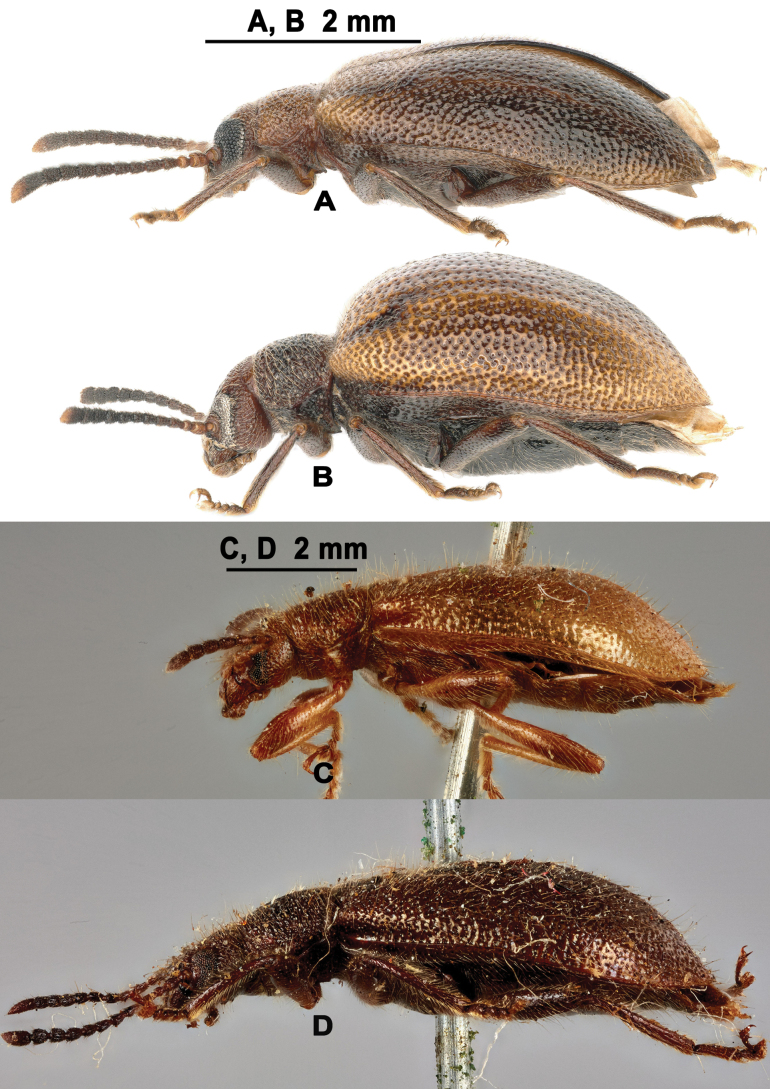
Lateral view of L. (Lagriella) spp. **A, B.**L. (Lagriella) cordata sp. nov.: **A.** Male; **B.** Female; **C.**L. (Lagriella) andrewesi, female; **D.**L. (Lagriella) mima, female. Scale bars: 2 mm (**A, B**); 2 mm (**C, D**).

**Figure 6.**
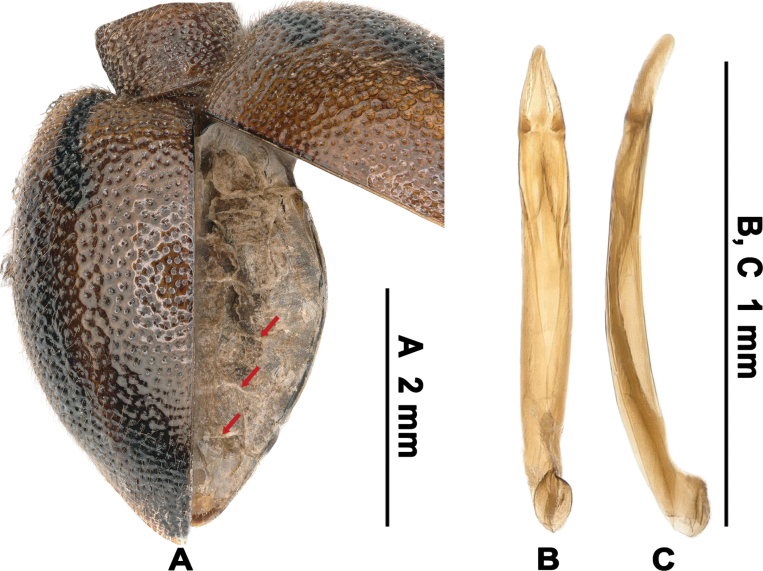
Partial dorsal surface and aedeagus of L. (Lagriella) cordata sp. nov. **A.** Partial dorsal surface (female, 2023.III.30, Guiyang City, Baiyun District, Niuchang Buyi Ethnic Town), showing exposed abdominal tergites when right elytron was lifted (arrow indicates posterior margin of tergites); **B, C.** Aedeagus: **B.** Ventral view; **C.** Lateral view. Scale bars: 2 mm (**A**); 1 mm (**B, C**).

(5 ♂ 8 ♀, all preserved in CNU, with the exception of the final female from Hong Kong). ***Holotype***: China • ♂ (Figs 4A, B, 5A); Guizhou, Guiyang City, Baiyun District, Niuchang Buyi Ethnic Town; 2024.V.22–24; Ri-Xin Jiang leg.; flight trap. ***Paratypes***: China • 1♂ 1♀ (Figs 4C, D, 5B); “广西贺州大桂山七星冲” [Guangxi: Hezhou City, Daguishan Mountain, Qixingchong]; 24°03'41"N, 111°39'50"E; alt. 100 m; 2021.V.9; “白兴龙采” [Xing-Long Bai leg.]; No. 21-5-9-1 • 3 ♀; “广西贺州大桂山留羊顶” [Guangxi, Hezhou City, Daguishan Mountain, Liuyangding]; 24°09'08"N, 111°43'03"E; alt. 660 m; 2021.V.9; “白兴龙采” [Xing-Long Bai leg.; No. 21-5-9-2 • 1 ♀: “广西大桂山森林公园” [Guangxi Dagui Mountain Forest Park]; 24°10'01"N, 111°43'16"E; alt. 160 m; 2021.V.10; “白兴龙采” [Xing-Long Bai leg.]; No. 21-5-10-1 • 1 ♂; same as the holotype • 1 ♂; same as the holotype, but 2024.V.15–21 • 1 ♂ 1 ♀ (Fig. 6A); Guizhou, Guiyang City, Baiyun District, Niuchang Buyi Ethnic Town; 2023.III.30; Ri-Xin Jiang leg. • 1 ♀; Guizhou, Guiyang City, Baiyun District, Niuchang Buyi Ethnic Town; 2024.I.13; Ri-Xin Jiang, Yong Zhou and Zhi-Xiang Tan leg. • 1 ♀; “湖南永州市江永县千家峒大泊水瀑布景区大门” [Hunan, Yongzhou City, Jiangyong County, Entrance of Qianjiadong Daboshui Waterfall Scenic Area]; 25°24'36"N, 111°18'41"E; alt. 330 m; 2020.VIII.28–29; “邱鹭采” [Lu Qiu leg.] • 1 ♀; “黃龍坑郊遊徑” [Hong Kong, Wong Lung Hang Country Trail]; alt. 460 m; 2023.VII.25; Matthew-T Hamer, Yuen-Lam Ng and Kit-Lam Tang leg.; litter sifting; IZCAS.

#### Diagnosis.

Body small-sized, elytra bearing a dark longitudinal band medial to humeral callosity (Fig. 4A, C), male hindwings well-developed, epipleura strongly widened (Fig. 4B, D), about 3× as wide as metanepisternum, pronotum transverse; female apterous, elytra exceptionally convex (Fig. 5B), abruptly dilated in basal 1/3 and forming a heart-shaped outline when closed (Fig. 4C). The new species resembles L. (Lagria) kondoi Masumoto, 1988 in the large eyes and transverse pronotum but differs in the following: 1) the shorter terminal antennomere, 2) the broader elytra, 3) the coarser punctation on pronotum and elytra, and 4) the apterous female (Masumoto 1988). Within its subgenus, the new species is most similar to L. (Lagriella) andrewesi, key diagnostic differences are provided in the key above.

#### Description.

***Holotype*** ♂ (Figs 4A, B, 5A). Body length 4.9 mm, width 2.6 mm. Body small-sized, hindwings well-developed, slightly shiny, about 1.88× as long as wide; entirely brown, except for darker antennomeres III–XI, elytral posterior half, metaventrite and abdominal ventrites, each elytron with a dark longitudinal band in impression between humeral callosity and elytral disc, arched inward, extending from elytral base to basal 1/4. Dorsal, ventral surface and legs with short setae.

Head circular, widest at eye level. Mouthparts slightly protruding forward; apical maxillary palpomeres campanulate, with oblique apical margin, cavate in apical surface; mandibles small, bending inward, embracing labrum; labrum slightly transverse, the visible portion cordiform, slightly emarginate anteriorly; labroepistomal membrane visible; epistoma elevated, positioned higher than labrum, strongly transverse, trapezoidal, with anterior margin nearly straight, sparsely covered by minute punctures. Frons densely and coarsely punctate, with anterior portion gently elevated, broadly separated from epistoma by forward-arched frontoepistomal impression; frontal canthus weak. Eyes large, slightly bulging, with anterior margin moderately emarginate, interocular distance about 0.74× as long as eye diameter. Antennae filiform, surpassing mesocoxae when directed backwards but not reaching metacoxae, length ratios of antennomeres I–XI as 19: 14: 20: 18: 16: 17: 19: 18: 18: 18: 33, antennomere XI elongated, slightly inflated distally, with pointed apex, almost equal to the combined length of two preceding antennomeres.

Prothorax transverse, about 0.57× as long as wide, widest across median portion, slightly wider than head at widest point, narrowly constricted before base. Pronotum convex, densely and coarsely punctate, denser in lateral portions, median intervals forming elevated, broad wrinkles, with disc transversely impressed in mid area before base; anterolateral angles obtuse, posterolateral angles round, slightly projecting laterally; anterior margin arched forward, posterior margin almost straight with narrow carina, lateral portions roundly bending toward ventral surface with margins invisible in dorsal view. Prosternal process narrow, not elevated, expanded backwards, apex round.

Scutellar shield triangular, punctate, with straight lateral sides, acute apex. Elytra wide, slightly convex (Fig. 5A), expanded in basal 1/2, then contracted backwards, 1.44× as long as wide and 6.03× as long as prothorax, with disc impressed medially in basal 1/4; surface with irregular and coarse punctures separated by interspaces equal as to 2× puncture diameter, punctures becoming sparser toward elytral apex, some elytral intervals forming elevated wrinkles, especially on anterolateral and anterior portions; humeral callosity prominent, punctate, rounded in dorsal view, separated from disc by shallow impression; elytral margins distinct, visible in dorsal view except for portions beneath humeral callosity; epipleura punctate, strongly widened, about 3× as wide as metanepisternum. Metaventrite sparsely punctate, emarginate apically, elevated, higher than metacoxae.

Legs short, unmodified; femora slightly clavate, punctate; tibiae straight; metatarsomere I longest, almost equal to the combined length of metatarsomeres II–IV. Abdominal ventrites sparsely punctate, each ventrite with two nearly round impression laterally. Aedeagus slightly curved in lateral view (Fig. 6C); parameres triangularly elongate, with lateral margins slightly curved inward distally (Fig. 6B).

***Female*** (Figs 4C, D, 5B). Body length 4.9 mm, width 2.9 mm. Body apterous (Fig. 6A, not described female), prothorax and legs darker, the elytral dark band darker and shorter, broadening posteriorly. Eyes small, interocular distance about 2.52× as long as eye diameter; antennae shorter, length ratios of antennomeres I–XI as 18: 10: 18: 16: 13: 14: 16: 15: 15: 13: 22, slightly shorter than the combined length of two preceding antennomeres; prothorax trapezoidal, about 0.62× as long as wide, widest before anterior margin, pronotum strongly convex; elytra exceptionally convex (Fig. 5B), sharply expanded in basal 1/3, then contracted backwards, 1.24× as long as wide, with disc not impressed medially in basal 1/4, humeral callosity not prominent, elytral margins visible in apical portions; epipleura wider. Metaventrite narrow.

#### Etymology.

The species epithet *cordata* (Latin for heart-shaped) refers to the female elytra forming a cordate outline when closed together.

#### Distribution.

China: Guizhou, Guangxi, Hong Kong, Hunan.

#### Ecology.

**Figure 7.**
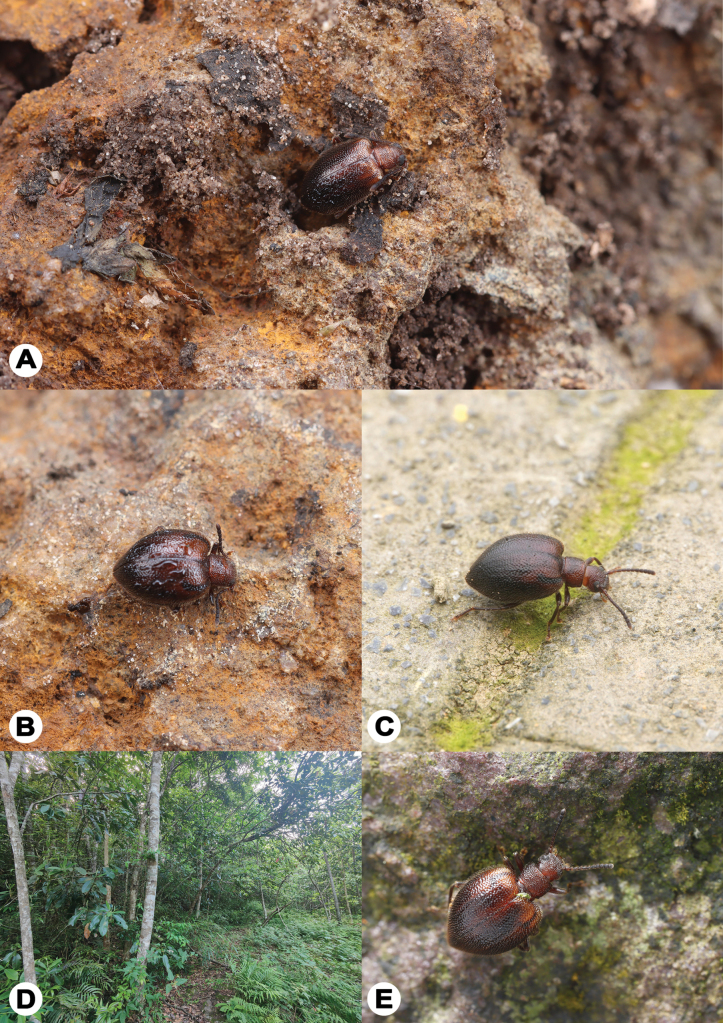
Natural habitats of L. (Lagriella) cordata sp. nov. **A, B.** Adult in overwintering state beneath rocks (note appendage retraction) (photographed by Dr Ri-Xin Jiang): **A.** Male (2023.III.30, Guizhou, Guiyang City, Baiyun District, Niuchang Buyi Ethnic Town); **B.** Female, same as the previous; **C.** A female wandering on artificial pavement (Guizhou, Zunyi, Dabanshui Forest Park, photographed by Mr Yu-Zhou Huang); **D.** General habitat in Guangxi, Jinxiu Yao Autonomous County, Dayao Mountain National Nature Reserve, photographed by Mr Qian-Le Lu; **E.** A female on rock surface (same data as D).

All examined female specimens were collected while actively moving from the ground. In contrast, some male specimens from Guiyang, Guizhou were captured using flight-intercept traps by Dr Ri-Xin Jiang, suggesting sexual dimorphism in wing morphology (female apterous, versus hindwings well-developed in male). Specimens from Guangxi, provided by Xing-Long Bai, included individuals attracted to light, indicating possible phototactic behaviour. Notably, three specimens collected in January and March in Guizhou were found beneath stones, suggesting the new species may utilize subterranean shelters for overwintering (Fig. 7A, B).

## Supplementary Material

XML Treatment for Lagriella

XML Treatment for Lagria (Lagriella) andrewesi

XML Treatment for Lagria (Lagriella) bimarginata

XML Treatment for Lagria (Lagriella) mima

XML Treatment for Lagria (Lagriella) cordata

## ﻿Additional information

### Use of AI

No use of AI was reported.

### Author ORCIDs

Yong Zhou https://orcid.org/0000-0002-1411-7563

Bin Chen https://orcid.org/0000-0002-5227-7736

### Data availability

All of the data that support the findings of this study are available in the main text.
